# Dentoskeletal effects of class II malocclusion treatment with the modified Twin Block appliance

**DOI:** 10.4317/jced.56241

**Published:** 2019-12-01

**Authors:** Shabnam Ajami, Anahita Morovvat, Bahar Khademi, Dana Jafarpour, Neda Babanouri

**Affiliations:** 1 Orthodontics Research Center, Department of Orthodontics, Shiraz University of Medical Science, Shiraz, Iran

## Abstract

**Background:**

The purpose of this study was to prospectively assess the dentoskeletal effect of a modified Twin Block appliance for treatment of class II malocclusions.

**Material and Methods:**

Lateral cephalograms of 25 Class II malocclusion patients were compared to evaluate skeletal, dentoalveolar and soft tissue changes pre- and post-treatment with a modified Twin Block appliance. A total of 33 angular and linear variables were used for analysis. The differences were calculated at the start and end of treatment. The paired T test was performed to compare the cephalometric measurements before and after treatment.

**Results:**

Compared the pre- and post- treatment measurements, there was a significant increase in SNB (*P*<0.001), CO-Gn (*P*<0.001), ANS-Me (*P*=0.001), Mandibular base (*P*<0.001), Lower 1 to NB (°) (*P*=0.004), Lower 1 to NB (mm) (*P*<0.001), and Z-angle (*P*=0.001) following functional therapy with modified Twin Block appliance. On the other hand, a significant decrease was observed in ANB (*P*<0.001), NA-Pog (*P*<0.001), overjet (*P*<0.001), and overbite (*P*=0.007), Upper 1 to palatal plane (*P*=0.007), UL-E-line (*P*<0.001), LL-E-line (*P*=0.001), and H-angle (*P*=0.002) after treatment with modified Twin Block appliance.

**Conclusions:**

The modified Twin-Block improves facial esthetics in Class II malocclusion by a combination of changes in skeletal as well as dentoalveolar structures. The increase of mandibular unit length was observed to be due to a true mandibular growth not just a repositioning of the mandible. The modified appliance, however, did not show any superior effects in terms of less dental compensation compared to the conventional Twin–Block appliance.

** Key words:**Dentoskeletal effect, Modified Twin Block, Class II malocclusion.

## Introduction

Class II malocclusion is considered as one of the most prevalent craniofacial deformities. The efficiency of mechanotherapies and the addressed treatments time in this group of malocclusions are controversial topics (1) since skeletal class II malocclusion may be the result of sagittal mandibular deficiency, maxillary excess or a combination of these two (2,3).

Functional orthopedic appliances which are the acceptable growth modification mechanotherapies in skeletal class II malocclusions may vary depending on their affected areas (2). In general it has been accepted that tissue-born appliances cause less dental compensation when compared with tooth-born appliances (2). Among tooth-born appliances, Twin Block is one of the most applied ones (4,5). Ease of acceptance by patients and gain of compliance have made this appliance become increasingly popular. In addition, the separate upper and lower units facilitate speech and mastication functions (5,6).

There are some controversies among studies investigating the effect of Twin Block on dental, skeletal and soft tissue components (5,7). While a number of studies have suggested an increase in the mandibular length, some researchers did not report such an increase (5). However, control of maxillary growth, enhancement of mandibular growth, and modification of dentoalveolar systems are the expected results following functional appliance therapies. Many investigations revealed that the most significant effects of functional therapies are the dentoalveolar modifications (8) since these appliances are supported by teeth rather than the maxillary and mandibular bone. Placement of functional appliances causes displacement of condyle in the glenoid fossa, and stimulates growth of mandibular growth sites meaning condylar cartilage, condylar neck area and ramus (9). The efficacy of treatment highly depends on the growth potential and response of condylar cartilage and other mandibular growth sites (9,10). Moreover, there is still controversy on whether growth modification actually increases the total mandibular length or it only increases the rate of the genetically expected amount of growth (9,11).

The results of the recent studies comparing bionator and Twin Block have shown comparable results between the two appliances regarding dentoalveolar and mandibular position; though Twin Block was more efficient in retarding the maxillary forward growth (8). Other studies have also showed a significant increase in the mandibular length using Twin Block (12). Since dental compensation in Twin Block has been proposed as one of the drawbacks of Twin Block appliances (12), the authors have decided to modify the appliance in a way that dental movements are the least and volunteer advance position of mandible by patients is greater than conventional Twin Blocks.

The purpose of this study was to retrospectively assess the dentoskeletal effects of a modified Twin Block appliance for treatment of class II malocclusions.

## Material and Methods

For this retrospective cross sectional study, the dental documents of patients treated in Shiraz School of Dentistry were investigated. The patients entered the study inquiring the following inclusion criteria: chronologic age of 8-12 years of old, having class II division I malocclusion, having at least an end-to-end molar relationship, an overjet between 5 and 10 mm, complete available treatment documents, at least 17 hours of appliance wear every day, and having normal or horizontal growth pattern. Also all the subjects should have had same bite recording technique including: one step mandibular enhancement, edge-to-edge incisors position and bite opening between 2 to 5 mm and they should have been treated using a modification of Twin Block.

In addition, the subjects demonstrating the following criteria were excluded from the study: maxillary prognathism, severe protrusion of maxillary incisors, severe dental crowding (space deficiency more than 4 mm), anterior dental open bite, previous orthodontic treatment, and extracted permanent teeth.

• The Appliance Design

The modified Twin Block, which was constructed for all the selected patients, demonstrated some variations in comparison to the conventional ones:

- All the lower incisors had the acrylic capping.

- The labial bow in the anterior lower segment was embedded in an acrylic bar.

- A wax relief was considered in the lingual side of the lower incisors.

- After two months of using the appliance, the retentive clasps were omitted from the lower part of the appliance and patients were asked to keep the lower removable plates in position voluntarily with muscular activities.

- In the upper arch, the jackscrew was expanded until over correction of buccal crossbite was achieved in a correct mandibular position.

• Data Entry

Finally 25 patients were evaluated for dental and skeletal effects. Lateral cephalometric radiographs from two time points were used: 1) the pretreatment examination; and 2) at completion of full appliance treatment (after 18 months). The cephalometric radiographs from all subjects were traced and the cephalometric data were measured. A total of 33 angular and linear variables were used in this analysis ( Table 1)

**Table 1:**
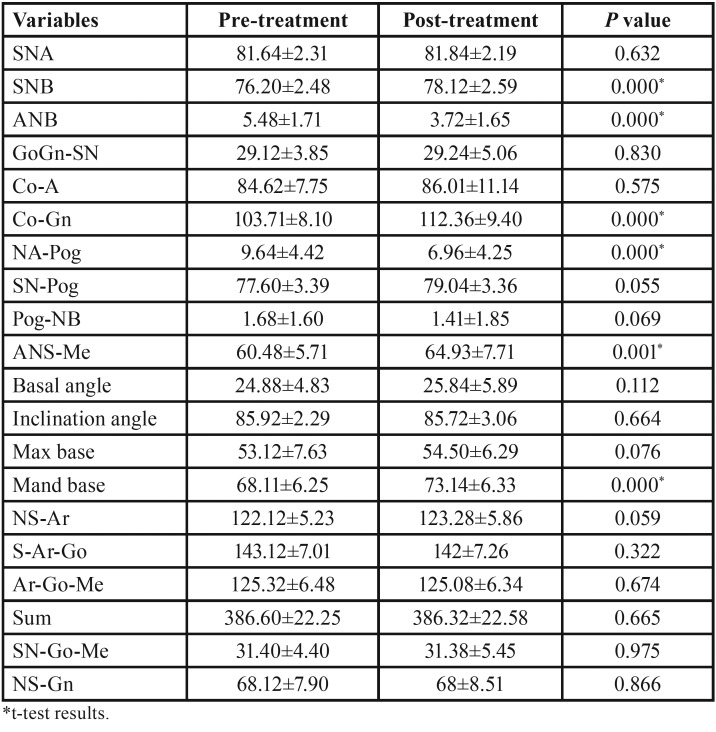
Descriptive information and paired t-test results of pretreatment and post-treatment for skeletal variables.

• Data Analysis

Data were analyzed using SPSS software version 22 (SPSS Inc, Chicago, IL, USA). The paired t-test was performed to compare the cephalometric measurements before and after treatment. *P* value of <0.5 was considered to indicate statistical significance.

## Results

In this study, treatment documents of 25 patients who were treated with modified Twin Block were evaluated before and after functional therapy.

The mean, standard deviations and the results of paired t-test for the pretreatment and post-treatment cephalometric measurements are presented in  Table 1- Table 3.

**Table 2:**
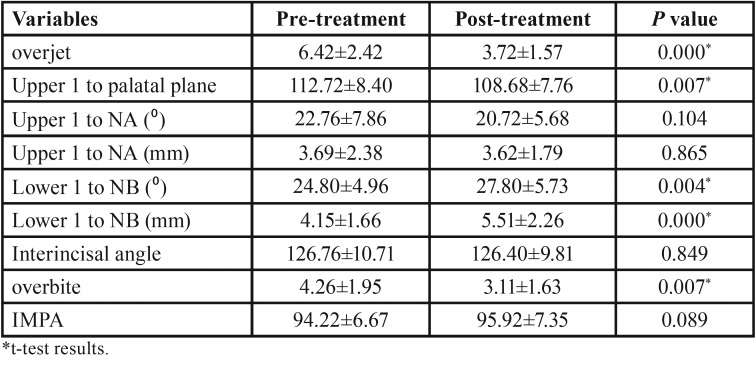
Descriptive information and paired t-test results of pretreatment and post-treatment for dentoalveolar variables.

**Table 3:**
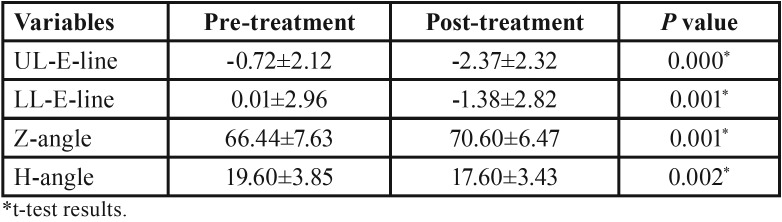
Descriptive information and paired t-test results of pretreatment and post-treatment for soft tissue variables.

As shown in  Table 1, there were a significant increase in SNB (*P*<0.001), CO-Gn (*P*<0.001), ANS-Me (*P*=0.001), Mandibular base (*P*<0.001), Lower 1 to NB (mm) (*P*=0.004), Lower 1 to NB (mm) (*P*<0.001), and Z-angle (*P*=0.001) following functional therapy with modified Twin Block appliance. On the other hand, a significant decrease were observed in ANB (*P*<0.001), NA-Pog (*P*<0.001), overjet (*P*<0.001), and overbite (*P*=0.007), Upper 1 to palatal plane (*P*=0.007), UL-E-line (*P*<0.001), LL-E-line (*P*=0.001), and H-angle (*P*=0.002) after treatment with modified Twin Block appliance.

## Discussion

Class II malocclusion can occur by a combination of skeletal, dentoalveolar and soft tissue changes. However, as Franchi *et al.* (13) has reported, most of the patients suffer from mandibular anteroposterior deficiency (14). Therefore, among various treatment strategies implemented to treat patients with class II malocclusions, functional appliance has been shown to be an ideal treatment plan in growing individuals. Since dental compensation in Twin Block has been proposed as one of the drawbacks of Twin Block appliances (12), the authors decided to modify the appliance in a way that dental movements are the least and volunteer advance position of mandible by patients is greater than conventional Twin Blocks. Therefore, the purpose of this study was to prospectively assess the dentoskeletal effects of a modified Twin Block appliance for treatment of class II malocclusions.

The results of this study showed that the modified Twin Block appliance can be used as a treatment option for Class II malocclusion by a combination of skeletal (forward shift of the mandible, increase in in SNB, mandibular unit length and mandibular base) and dental effects (maxillary incisor retroclincation and mandibular incisors proclination).

-Skeletal effects

It has been shown that the stretch of the muscles and the adjacent soft tissues of the facial skeleton cause the repositioning of the forwardly shifted mandible to its original place, leading to a reciprocal restrictive effect on the maxilla that is known as headgear effect (14,15). Previously, several studied have reported such effect on maxilla with a Twin Block appliance. As O’Brien *et al.* (16) have observed, 13% of overall skeletal changes was attributed to the restraining effect on maxillary growth with the Twin Block appliance. In addition, Illing *et al.* (17) has confirmed a small mean reduction in SNA angle. However, a number of other studies have not found any significant orthopedic effect on the maxilla using a Twin Block (14,18). Similar to the results of Khoja *et al.* (5) who reported no statistically significant change in SNA angle and maxillary unit length, the findings of the present study showed no statistically significant reduction in SNA angle. Moreover, the alteration of maxilla Co-A was also not significant in our study.

In the literature, there is controversy over the effects of functional appliance on mandibular growth. A number of studies have advocated that functional appliance can lead to the anterior repositioning of point B and pogonion, causing an increase in the SNB angle (17,19). Similarly, Baysal and Uysal. (4) reported a significant increase in SNB angle following Twin Block application. Khoja *et al.* (5) also found a significant increase in SNB angle by 1.56˚ and mandibular unit length of 3.27 mm over a 12-month period. Moreover, Illing *et al.* (17) and Toth and McNamara (20) found an increase in mandibular unit length (Co-Gn) when compared with the controls. The results of the present study are in line with previous works. As the findings revealed, a significant increase occurred in the SNB angle as well as the mandibular unit length (Co-Gn) following the modified Twin Block appliance. Also, a significant increase of 5.03mm was observed in the mandibular base showing that the increase of mandibular unit length (Co-Gn) was because of a true mandibular growth not just a repositioning of the mandible.

Previous researches have also found that a decrease in SNA, an increase in SNB, or a combination of these may result in the reduction of ANB angle following Twin Block appliance therapy. In a study by Toth and McNamara (20), a reduction in ANB angle by 1.8˚ was reported in patients who received Twin Block appliance. Furthermore, Illing *et al.* (17) observed a statistically significant reduction in ANB angle. Similar to the above-mentioned works, our findings indicated a mean reduction in ANB angle by 1.76˚ following modified Twin Block appliance therapy, being mainly due to an increase in the SNB angle.

-Dentoalveolar effects

In a study by Illing *et al.* (17), the researchers found a more pronounced reduction in the inclination of maxillary incisors in the Twin Block group compared to Bass and bionator. The effect was greater by incorporation of a labial bow into the appliance. In addition, O’Brien *et al.* (16) observed a maxillary incisor retraction which led to a significant overjet reduction. Therefore, the authors proposed that Class II malocclusion was mostly corrected by dentoalveolar movements rather than mandibular growth. In another work by Khoja *et al.* (5), a significant retroclination of maxillary incisors was reported following Twin Block therapy. Likewise, in our study, a significant reduction in the upper 1 to palatal plane occurred, showing a significant retrusion of maxillary incisors following modified Twin Block appliance therapy.

The effect of Twin Block appliance on mandibular incisors has been found to be controversial in literature. While Illing *et al.* (17) reported no significant change in mandibular incisor inclination following Twin Block application, Lund and Sandler (21) and Khoja *et al.* (5) observed a statistically signifcant increase in mandibular incisor inclination.. In line with the works of Lund and Sandler (21) and Khoja *et al.* (5), we found a statistically significant increase in Lower 1 to NB. The significant retrusion of maxillary incisors along with the significant increase in the mandibular incisor inclination led to a significant overjet reduction (*P*<0.001) and, thus, limited the potential for further growth.

-Soft tissue changes

The effect of Twin Block appliance on soft tissue is also debated in the literature. While Quintao *et al.* (22) and Khoja *et al.* (5) demonstrated a significant change in upper lip position due to maxillary incisor retrusion following functional appliance treatment, Morris *et al.* (23) reported no significant change in the sagittal position of upper lip regardless of great overjet reductions. In the present study, upper lip became significantly less bulged out in the patient underdone modified Twin Block therapy. On the other hand, while in the study of Baysal and Uysal (4) and Khoja *et al.* (5), greater progression of the lower lip, lower lip sulcus and soft tissue pogonion was observed in the Twin Block group, Quintao *et al.* (22), did not find any significant changes in any of the lower lip variables. In our study, lower lip changes were observed with LL-E-line being significantly reduced (*P*=0.001).

In a study by Varlik *et al.* (7), the significant increase found in Z-angle in patients treated with the Twin Block appliance was attributed to the forward movement of soft tissue of chin. Furthermore, Khoja *et al.* (5) also reported a significant increase in Z-angle following the Twin Block therapy. Our results validate those findings as a significant increase (*P*=0.001) was observed following modified Twin Block application.

Regarding the H-angle, Holdaway (24) showed that the angle decreases as the facial convexity decreases. In addition, Baysal and Uysal (4) found a significant reduction in the H-angle in their study, which showed an improvement in the facial convexity following Twin Block application. Moreover, Khoja and colleagues (5) detected a significant reduction in this angle. The authors attributed this finding to the combination of upper lip retraction and forward movement of the soft tissue pogonion. In our study, we also found a significant decrease in this angle (*P*=0.002).

-Limitations

The present research assessed the effects of a modified Twin Block appliance over a period of 18 months. Therefore, it is suggested that further studies be carried out to evaluate the long term effects of the modified Twin Block appliance on mandibular growth. The other limitation of this study was the absence of the control group which treated with the conventional Twin Block appliance. The authors proposed prospective clinical trial study on this subject for the future study.

## Conclusions

The results of the present study suggested that the modified Twin-Block improves facial esthetics in Class II malocclusion by a combination of changes in skeletal as well as dentoalveolar structures. The increase of mandibular unit length was observed to be due to a true mandibular growth not just a repositioning of the mandible. The modified appliance, however, did not show any superior effects in terms of less dental compensation compared to the conventional Twin Block appliance.
